# A fragmented fish community: evaluation of the present distribution and drivers of fish communities in the lower uThukela River, KwaZulu-Natal, South Africa

**DOI:** 10.1007/s10641-026-01822-y

**Published:** 2026-03-04

**Authors:** Bradley van Zyl, Matthew J. Burnett, Celine Hanzen, Colleen T. Downs

**Affiliations:** 1 Centre for Functional Biodiversity, Discipline of Biological Sciences, University of KwaZulu-Natal, Private Bag X01, Scottsville, Pietermaritzburg, KwaZulu-Natal 3209 South Africa; 2 Healthy Ecosystems, Institute of Natural Resources NPC, P.O. Box 100 396, Scottsville, 3209 South Africa

**Keywords:** Instream barrier, Fishway, River connectivity, Fish migration, Freshwater ecology, Environmental variables, Diadromy

## Abstract

In South Africa, a water-scarce country, the construction of dams and weirs is increasing to ensure water security for a growing nation. The human need for this infrastructure is generally prioritised over its environmental impact, particularly for aquatic fauna with migratory needs. Such infrastructure can block migratory pathways for fish, trap sediment, and alter river flow regimes. The Lower Thukela Bulk Water Supply Scheme (LTBWSS) abstraction weir was constructed on the lower uThukela River in KwaZulu-Natal Province, South Africa, in 2017, near the town of Mandini. The riverine stretch where it is constructed is a critical interface for marine, estuarine, and freshwater migrant local fish species. As such, a vertical-slot fishway was incorporated into its design to facilitate the movement of fish over the weir. This study utilised data collected from field surveys at three sites upstream of the weir, four sites downstream of it, and two sites on the Mandini Stream, a tributary of the uThukela, to assess the impact of the weir on local fish community structures. Multivariate statistical analyses were employed to identify the environmental variables influencing fish community structures and select fish species in the region. The results showed that the dominant cover types, dominant substrate, average depth, and water temperature significantly affected fish communities in the study. Individual species were driven by different habitats, water velocity-depth profiles, and physical water-quality variables. Furthermore, the presence of the LTBWSS has influenced three cichlid species, particularly *Tilapia sparrmanii,* which was previously shown to favour silt substrate. The resulting loss of downstream sedimentation because of the LTBWSS weir has led to the absence of the species, along with synergistic effects on water quality and quantity in the downstream sections of the uThukela River.

**Supplementary information:**

The online version contains supplementary material available at 10.1007/s10641-026-01822-y.

## Introduction

Freshwater ecosystems provide essential ecosystem services important for human livelihoods and maintain environmental integrity (Hanna et al. 2018), such as drinking water, controlling natural water quality, supplying food, facilitating habitat for organisms, regulating the climate, and creating recreation and tourism options, to name a few, and are often irreplaceable (Postel and Carpenter 1997; Hanna et al. 2018; Kaval 2019). Their societal and ecological value is high, particularly in developing regions where the sustenance of many people depends on these services (King and Pienaar 2011; Fouchy et al. 2019). As a result of this dependence, freshwater ecosystems are among the most threatened ecosystems on the planet, often severely modified through anthropogenic development to meet the growing needs of the human population (Dudgeon et al. 2006; Rodell et al. 2018; Du Plessis 2019). Such developments often excessively use water resources to support essential societal services, contributing to biodiversity loss worldwide (Dudgeon 2010; Dugan et al. 2010). This is often caused by dams, weirs, and other instream barriers constructed for water provision to various economic sectors, flood control, flow alterations, and hydropower generation (Belletti et al. 2020). The use of water across sectors when building instream structures contributes to river fragmentation and associated habitat loss and habitat alterations (World Commission on Dams 2000; Carpenter et al. 2011; Fouchy et al. 2019; Barbarossa et al. 2020), shifts in hydrological dynamics (World Commission on Dams 2000; Zuo and Liang 2015).

Biodiversity is negatively impacted by instream structures; however, in addition, additional water quality and pollution issues resulting from intensified land-use and agricultural practices, as well as the introduction of alien invasive species, can compound the stressors associated with instream barriers and vice *versa* (Evans et al. 2019). Translocated invasive species threaten indigenous biodiversity by predating on or competing with indigenous biota for resources, diminishing or completely eradicating resident populations (Ellender and Weyl 2014; Gallardo et al. 2016; Burnett et al. 2023). Climate change exacerbates the detrimental effects of these stressors (Denicola et al. 2015; Mittal et al. 2016).

Fish form essential constituents of river ecosystems and play a major role in ecosystem health (Fausch et al. 2002; Dugan et al. 2010). Freshwater fish populations support various ecological functions, including nutrient transport between habitats, pest control, sediment bioturbation, and food production (Holmlund and Hammer 1999). In addition, most fish migrate to some degree, allowing them to occupy several ecological niches throughout their lifetimes (Lucas and Baras 2008). As a result, they act as agents for the cycling of nutrients and energy between the aquatic environments they inhabit, including those between saltwater and freshwater ecosystems (McIntyre et al. 2016). These migrations occur for various reasons, such as spawning, feeding, and evading predators or harmful environmental conditions, but are ultimately important to individual and population health (Northcote 1978; Lucas and Baras 2008; McIntyre et al. 2016). Through their various ecological functions, certain fish species are often valuable indicators of ecosystem health (Chovanec et al. 2003). Migratory species are generally better indicators because of their mobility, unique habitat needs, and vulnerability to a variety of stressors and resource degradation (Harris 1995; Dugan et al. 2010).

Knowledge of African freshwater fish biology and ecology is sparse (Weyl and Chakona 2020), which is a concern when facing increasing anthropogenic stressors that alter environmental conditions causing local extinctions (Tickner et al. 2020; Burnett et al. 2023), or implementing mitigation measures against anthropogenic structures, for example; fishways are potentially poorly designed because of inadequate understanding of fish responses or their environmental requirements (Silva et al. 2018). Therefore, to effectively manage fish populations and their ecosystems, it is essential to understand the environmental drivers of fish species’ community structure and distribution within a particular system (Weyl and Chakona 2020; Desai et al. 2021; Evans et al. 2022).

**Fig. 1:**
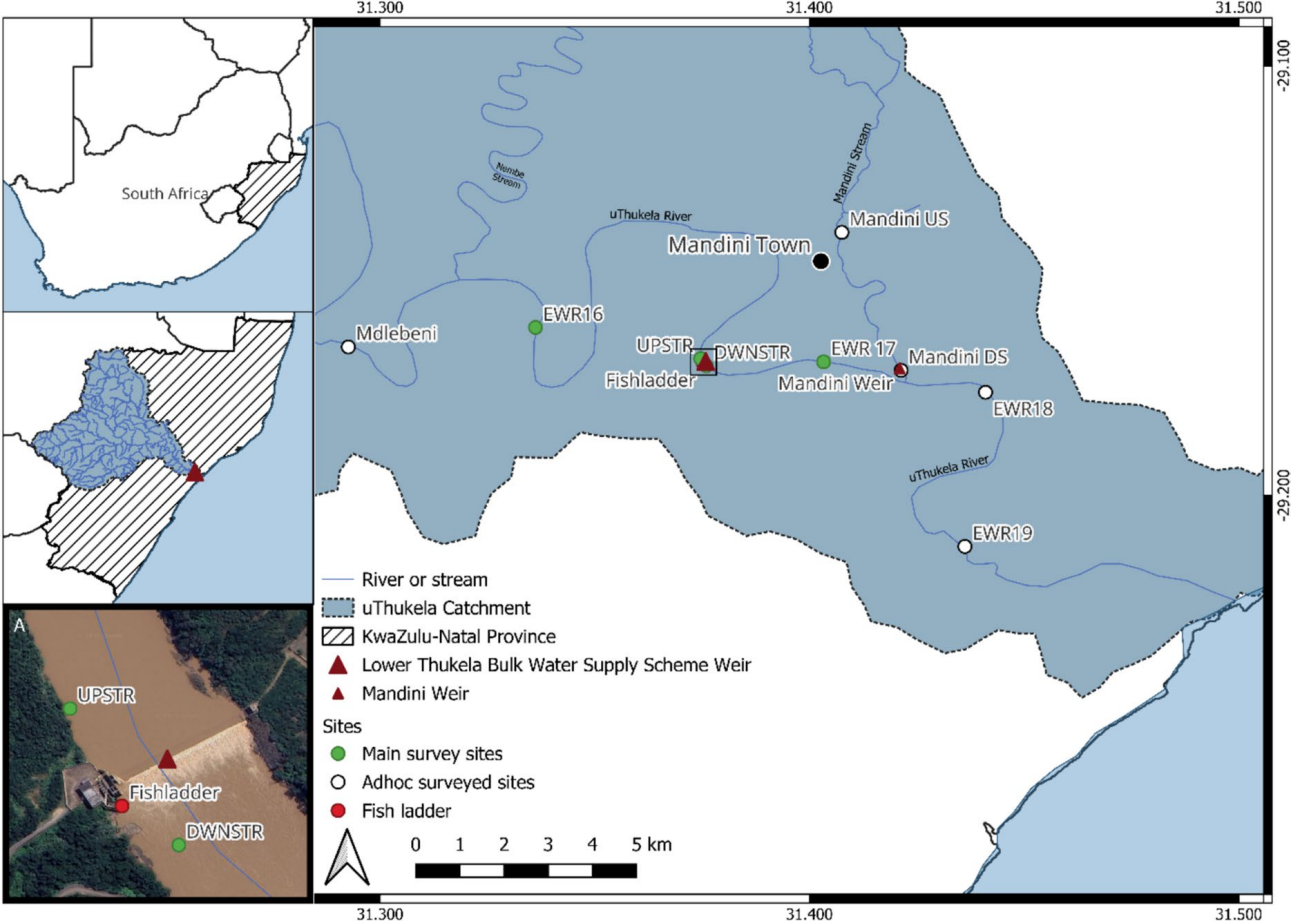
Map of the present study area, with sampling sites in the lower uThukela River, KwaZulu-Natal Province, South Africa. (Note: Insert A details sites around the Lower Thukela Bulk Water Supply Scheme)

The uThukela River is ~ 500 km long and has a catchment area of just under 30,000 km^2^, and it is South Africa’s second-largest river by volume and the largest catchment in KwaZulu-Natal Province (Department of Water Affairs and Forestry 2004; Fig. 1). Its source is in the Drakensberg Mountains, from whence it runs across central KwaZulu-Natal in an easterly direction until emptying into the Indian Ocean through the uThukela Estuary (Department of Water Affairs and Forestry 2004). Throughout its catchment, including its reach into the Indian Ocean, the uThukela River provides essential ecosystem services and resources to both humans and the environment (Department of Water Affairs and Forestry 2004; Department of Water Affairs 2013). Its water supply is valued not only by communities in its catchment but also by other areas in the country that receive water through its inter-basin water transfer schemes, including Gauteng Province, the economic hub of South Africa (Van Vuuren 2008; Department of Water Affairs 2013). The nutrients provided by the uThukela River are important to the varying ecosystems within its catchment, as well as the offshore marine environment, particularly the Thukela Banks and uThukela Marine Protected Area (De Lecea and Cooper 2016; Department of Environmental Affairs 2019; Wade et al. 2021). However, several water quality, quantity, habitat-altering, and wildlife disturbance stressors have resulted from the heavy use of rivers in the uThukela catchment, impacting both social and biological elements of the system (Department of Water Affairs and Forestry 2004; Department of Water and Sanitation 2022a). The uThukela River main stem and its tributaries have been impacted by water abstraction, effluent discharge, and various other pollution sources from processes associated with human settlements, sewerage treatment plants, factories, processing plants, and agricultural practices (Department of Water Affairs 2013; Umgeni Water 2017). Multiple stressors, caused by excessive water use and poor land management practices in the uThukela catchment, impact the system (Department of Water Affairs and Forestry 2004; Wade et al. 2021; Department of Water and Sanitation 2022a).

Water quality and quantity issues are major stressors in the lower uThukela catchment, with sources such as the Sappi Tugela Pulp and Paper Mill (hereon referred to as the Paper Mill) responsible for elevated salts and organics, the localised industrial, urban, and peri-urban areas contributing to toxicants, and the stock farms, sewerage plants, upstream sources, and sugarcane (*Saccharum officinarum*) farms contributing to organic contaminants and nutrient enrichment (Wade et al. 2021; Evans et al. 2022). Due to upstream water extraction through dams and river diversions, the natural flow regimes of the river have been disrupted, causing water quantity problems (Wade et al. 2021). Sand mining operations, indirectly triggered by lower flows in the region, have been linked to habitat stressors (Wade et al. 2021). These types of operations are known to alter the natural transport and deposition processes of riverine sediments (Koehnken et al. 2020). Unmanaged fish harvesting and the presence of non-native aquatic fauna in the area are stressors that disturb wildlife in the lower uThukela River, particularly at its mouth (Wade et al. 2021). Recent evaluations of the state of the lower uThukela River and estuary have classified it as an ecological category C (Wade et al. 2021; Evans et al. 2022), indicating that it is acceptable to be moderately modified (Kleynhans 1999). This shows that the fundamental ecosystem functions are largely unchanged, despite some loss of natural habitats and biota (Kleynhans and Louw 2007).

In 2017, the Lower Thukela Bulk Water Supply Scheme (LTBWSS) infrastructure in Mandini, KwaZulu-Natal, was commissioned to abstract water from the uThukela River to supply the KwaDukuza and Mandini local municipalities (Department of Water and Sanitation 2022b). Presently, it has the capacity to abstract 55 Ml/d of environmental water, with a future phase ultimately allowing the abstraction of up to 110 Ml/d (DWS 2022a). Abstraction rates of 55 Ml/d and 110 Ml/d translate into losses of river flow of 0.64 m^3^/s and 1.27 m^3^/s, respectively (DWS 2022a). The quantity and timing of these flows must meet ecological flow requirements, with the LTBWSS weir equipped with a vertical-slot fish ladder and a rock ramp to mitigate the loss of river connectivity for aquatic fauna (DWS 2022a; Burnett et al. 2024).

Our study aimed to determine the impact of the newly constructed LTBWSS as a barrier to migratory fish and the potential effects this may have on the local fish community structures in the lower uThukela River, KwaZulu-Natal, South Africa. Our objectives were to (1) determine the environmental drivers influencing fish community structures in the study area using measured instream hydraulic variables and habitat characteristics, and (2) determine the potential impact that the recently constructed LTBWSS weir has on the local fish community structures in the lower uThukela River.

## Methods

### Study area

The present study area focused on the lower parts of the freshwater portion of the uThukela catchment in KwaZulu-Natal Province, South Africa, near the town of Mandini. The study occurred in quaternary catchment V50C and V50D of the Department of Water and Sanitation (DWS) catchment management areas of South Africa, from the upstream site at Mdlebeni road bridge to the downstream site at the N2 road bridge (site EWR19; Fig. 1) on the uThukela River, which included the Mandini Stream that flows into the uThukela River (Fig. 1). The upper tidal limit of the uThukela Estuary penetrates approximately 8 km upstream from the mouth, located near site EWR19 (Fig. 1). Despite the study being in the freshwater reaches, the presence of diadromous fish was expected as some species in the region have dependencies on both freshwater and saltwater at different stages of their life cycles (Whitfield 1998; 2019; Skelton 2025).

The LTBWSS Weir was commissioned in 2017 and fitted with a vertical-slot fish ladder as a fishway. This, however, is designed to facilitate upstream migration of fish, and its efficacy in South African species is poorly understood (Burnett et al. 2024). The LTBWSS fish ladder is one metre wide and two metres high, with its upstream orifice located in the fine sediment trap water abstraction bay. It features fish screens to prevent fish from being pumped out of the river. The downstream migration route is considered to be over the top of the weir, following downstream flows.

Initially, we selected four sites to assess the fish community structures in the area surrounding the LTBWSS weir, with two sites upstream (sites EWR16 and UPSTR; Fig. 1) of the weir and two downstream of it (sites DWNSTR and EWR17; Fig. 1). One upstream and one downstream site were based on historical Environmental Water Requirement (EWR) sites. The other two were selected from the immediate upstream and downstream vicinity of the weir, with accessibility considered at all sites. The immediate upstream and downstream sites were deemed representative of a stretch of the river under the influence of the weir. In addition, additional ad hoc sites were included in the study to provide a comprehensive overview of fish communities in the lower uThukela River. These were one site further upstream (site Mdlebeni; Fig. 1), two sites further downstream on the uThukela River (sites EWR18 and EWR19; Fig. 1), and two sites situated on the Mandini Stream (sites Mandini US and Mandini DS; Fig. 1), a tributary which enters the uThukela River downstream of the LTBWSS weir. The downstream site on the Mandini (shown as Mandini Weir at Mandini DS site, Fig. 1) is situated below a natural waterfall with a weir built on top of it (barrier height is ca. 10 m), ~ 200 m upstream of its confluence with the uThukela River (Wade et al. 2021).

At each site, we selected the present biotope types for sampling, namely rapids, runs, glides, and pools, to ensure representative sampling of the site. Biotopes were descriptively identified using Wadeson and Rowntree (1998) and were determined by examining surface flows and water depth. Instream measurements were collected for each sampling effort within the biotope to determine velocity-depth classes and associated substrates and cover features. Different fish capture (fyke net, seine net, and electrofishing) methods were used to sample different velocity depth classes. Biotopes sampled varied according to the availability at the site during the sampling period.

### Fish capture

We conducted three fish collection surveys in May, June, and August 2021, as well as in February 2022, at sites located around the LTBWSS weir (Fig. 1; Table 1). Surveys were conducted seasonally, including two low-flow surveys and one high-flow survey. A second high-flow survey at selected sites (Table 1) in early 2022 could not be conducted because the uThukela River was in flood following a sustained high rainfall period from January to April 2022. Seine netting was possible at ad hoc sites, with limited electroshocking at site EWR18 (Table 1). Fish data obtained from additional ad hoc surveys in the lower uThukela basin were also used as part of the fish community assessment (Table 1). Fish collection techniques included fyke nets, electrofishing, and seine netting. Approval to conduct these surveys was granted by the conservation authority Ezemvelo KZN Wildlife (permit numbers OP1060-2021 and OP1060-2022). We had ethical clearance from the University of KwaZulu-Natal Animal Ethics Committee (permit number AREC/023/020).

**Table 1:** Fish collection dates per site and methods used in the present study.

Site	20–23 May 2021	15–17 Jun 2021*	23–31 Aug 2021	3–6 Dec 2021	27 Feb–2 Mar 2022*
Mdlebeni*	-	-	-	-	S
EWR16	F, E	-	F, E	Flood	Flood
UPSTR	F	-	F	F	Flood
DWNSTR	F, E	E	F, E	F, E	Flood
EWR17	F, E	-	F, E	F, E	S
EWR18*	-	-	S	-	E, S
EWR19*	-	-	S	-	S
Mandini US*	-	-	E	-	E
Mandini DS*	-	-	E	-	E

^*^Ad hoc sites and surveys

*F* fyke net, *E* electroshocker, *S* seine net

For the initial sites, we used a minimum of four small double-ended Dutch-type commercial fyke nets (T&L Netmaking, Mooroolbark, Australia) during a single overnight sample period at each site per survey. The fyke nets used were double-ended Dutch-type fyke nets with a 9.7 m leader net between the hoop nets, which were 600 mm high and 800 mm wide. The netting material for the entire net had a stretched mesh size of 20 mm. As a precautionary measure, otter guards were placed at the entrances of all nets to prevent their capture. We deployed nets using an inflatable boat powered by a small battery-operated sneaker motor (Watersnake Venom, Watersnake Electric Motors and Accessories, Australia). We first fastened the net to the bank, set the first fyke, and moved into the river at a ca. 30° angle to the bank in the direction of the water flow to set the other end of the fyke in the water. The fyke nets were set between 15:00 and 17:00 on the first day, then checked and removed between 8:00 and 10:00 the following day. During the May 2021 survey, a minimum of seven nets were used at the site immediately downstream of the weir to increase the sample size collected for another study that required fish for a catch-mark-recapture study. These nets were deployed for four days, with deployment between 17:00 and 17:30 on the first day. They were then checked in the morning between 10:00 and 11:00 and again between 17:00 and 18:00, continuing until the end of the survey. On the day of net removal at all sites, additional fish collection was conducted using electrofishing or seine netting, as applicable. By combining all three sampling techniques, an attempt was made to sample all accessible velocity-depth classes (Kleynhans 2007) at each site.

At all sites, except in the impoundment formed by the LTBWSS at the site directly upstream of the weir, we used a backpack-mounted SAMUS electrofisher (SAMUS 725MS Electrofisher, SAMUS Special Electronics, Poland). Electrofishing was conducted in flowing water less than a metre deep or slow-flowing water containing large amounts of debris or rocks that would make seine netting impossible. We adjusted the electrofisher’s current to match the conditions in each sample area, thereby optimising capture of a variety of fish species. We conducted electrofishing in teams of three, walking in the water with insulated waders. One person operated the electrofisher, and two people handled the landing nets on either side of the operator. Each effort was approached from downstream, moving up, allowing stunned fish to drift downstream towards the samplers. A series of 5-s bursts of electricity over a 2–5-min period was applied to each effort. A site was intermittently electrofished for no longer than 60 min. The landing nets used had a minimum mesh size of 3 mm and a maximum mesh size of 10 mm.

We conducted seine netting in the slow-to-no-flowing sections of the river, which were generally deeper, or the sections containing large shallow sand banks. We used two different seine nets where appropriate. The small seine net (length = 12 m, depth = 1.5 m, mesh size = 3 mm; Eigevis Group of Companies, Cape Town, South Africa) was used in areas where the water was shallow, slow to no flow, and had a relatively smaller sample area. We used the large seine net (length = 32 m, depth = 2.5 m, mesh size = 25 mm; Eigevis Group of Companies, Cape Town, South Africa) in slightly faster-moving water, where the small seine net had too much resistance or was more efficient in performing one large seine of an area. We performed a maximum of five seine net drags (each drag was recorded as an effort) per site. The ad hoc surveys used the same electrofishing or seine netting methods described above.

We placed captured fish per effort into a holding container filled with river water, then released them after measurements were recorded and all the site sampling was completed. Data recorded included counting, identifying the individuals to the species level, and measuring standard lengths to the nearest millimetre. Measurements of individually collected fish are useful for analysing population structure, as their size classes can be valuable indicators of the state of fish populations (Evans et al. 2022). Fish were released as close to the capture point as possible.

### Habitat assessment

We collected in situ physicochemical characteristics at each sampled site. We measured water-quality variables once per sample effort for the entire site using a calibrated XS Instruments PC 5 Tester multiparameter tool (XS Instruments, Italy). Measured water-quality variables included temperature, pH, total dissolved solids, electrical conductivity, and salinity. We determined the available habitat and velocity-depth profiles at five points within each effort conducted at the study site.

Each effort represented a biotope type and was assessed for its available habitat despite the biotope guiding the sampling process. For each effort, five data points were selected at random in the sampled effort. Habitat was visually assessed for substrate and cover features. We described substrate as either bedrock, boulders, cobbles, gravel, sand, mud, silt, or other (Kleynhans 1999, 2007). Cover features were described as either the substrate itself, position in the water column, marginal vegetation, overhanging vegetation, instream/aquatic vegetation, root wads, woody debris, undercut banks, depth, or rippled surface (Kleynhans 1999, 2007). The velocity depth profiles for each of the five points per effort were measured using a transparent Velocity Head Rod (GroundTruth, Hilton, South Africa). We determined velocity-depth classes following James and King (2010), using two velocity classes which represent suitability criteria for fish (Kleynhans 2007). We determined velocity depth classes in terms of two velocity classes: slow (< 0.3 m s^−1^) and fast (> 0.3 m s^−1^), with three depth classes in the slow category and four in the fast category. These are described as slow-very shallow (0.1 m); slow-shallow (0.1–0.5 m); slow-deep (> 0.5 m) and fast-very shallow (< 0.1 m); fast-shallow (0.1–0.2 m); fast-intermediate (0.2–0.3 m); and fast-deep (> 0.3 m). To determine the ecohydraulic flow classes, we used the average depth and average velocity for that effort.

We obtained historical (prior to the construction of the uThukela Weir) fish assemblages in the lower uThukela River from available literature on the area (Wade et al. 2021; Evans et al. 2022; Skelton 2025), as well as databases such as the Present Ecological State, Ecological Importance and Ecological Sensitivity (PESEIS) (Department of Water and Sanitation 2014), and the Freshwater Biodiversity Information System (FBIS) (FBIS 2022). Historical data provided a reference species list of occurrences for the sampled sites, allowing determination of which species were not collected in the present study.

### Analyses

To assess the relationships between fish communities and environmental factors in the lower uThukela River, we employed a range of statistical methods. All statistical analyses were performed with R version 4.2.1 (R Core Team 2022). A modelling approach using multivariate generalised linear models (Warton 2011) was used. Firstly, a collinearity check was performed on the environmental variables using Pearson’s test to ensure that the variables used in the model did not exhibit high correlations that could influence model accuracy. We then fitted fish species presence data to environmental variables to determine the environmental factors influencing community structure in the lower uThukela River using multivariate analysis of relative fish community abundances with the ‘mvabund’ package (Wang et al. 2012). We used the Wald Test with 250 resampling iterations through PIT-trap resampling (Warton et al. 2017). The effects of the tested environmental variables were considered to be significant at *P* < 0.05. An additional summary of the multivariate Generalised Linear Model (GLM) with 999 PIT-trap resampling iterations, using the Wald test and a significance level of *P* < 0.05, allowed us to further analyse the levels of the tested environmental conditions and understand predictions. Here, we used a ‘Gaussian’ family because of a high mean–variance and nonconvergence using ‘Poisson’ and ‘negative binomial’ families of GLMs. We saw this as sufficient to understand the linear regression relationship, understanding that predicted negative numbers are not relevant to our data set.

## Results

### Habitat

The dominant habitat variables relating to ecohydraulic flow classes, substrate, and cover features for each site in our study area are summarised in Table 2 using collated totals from all efforts conducted at each site. Secondary characteristics per site are also given to understand habitat availability. For the seven sites on the lower uThukela River, the slow-deep ecohydraulic flow class was characteristic for all but site EWR16, which had a slow-shallow classification. Sand was the dominant substrate across all sites on the lower uThukela, except at the UPSTR site, which had mud as the dominant substrate. Depth as a cover feature was dominant across all sites on the uThukela, with additional cover features at Mdlebeni and EWR17 consisting of marginal vegetation, at EWR16 consisting of a cobble substrate, and at the downstream site consisting of a boulder substrate. The two Mandini sites were characterised by slow-shallow ecohydraulic flow (Table 2).

**Table 2:** Summary of the dominant ecohydraulic flow class, substrate, and cover features of the various sites used for sampling in the lower uThukela River, KwaZulu-Natal, South Africa (see Fig. 1 for site locations)

Site	Distance from estuary limit (km)	Ecohydraulic flow class	Substrate	Cover
Mdlebeni	32.8	Slow-deep	Mud/sand	Depth/marginal vegetation
EWR16	24.7	Slow-shallow	Cobble/sand	Depth/cobble substrate
UPSTR	10.6	Slow-deep	Mud	Depth
DWNSTR	10.6	Slow-deep	Boulder/sand	Depth/boulder substrate
EWR17	7	Slow-deep/slow-shallow	Mud/sand	Depth/marginal vegetation
EWR18	4.11	Slow-deep/slow-shallow	Sand	Depth
EWR19	−1.8	Slow-deep	Sand	Depth
Mandini US	11.5	Slow-shallow	Bedrock/sand	Overhanging vegetation
Mandini DS	6.5	Slow-shallow	Mud/boulder	Depth/boulder substrate

### Fish species community composition

Historical survey data showed that a combined 24 indigenous freshwater fish species and two additional invasive species were present and collected in these Quaternary catchments (Department of Water and Sanitation 2014; Evans et al. 2022; FBIS, 2022) (Table 3). Furthermore, these data showed that of the 14 species found upstream of the weir, two were euryhaline, *Awaous aeneofuscus* (W.K.H. Peters, 1852) and *Eleotris fusca* (J.R. Forster, 1801). Species with expected distribution ranges in the study area added a further ten indigenous species and one invasive species to the expected species list (Skelton 2025; Table 3). We collected a total of 1251 fish from 23 identifiable (and two unidentifiable juvenile) species in the study area (Table 4) using fyke nets, electrofishing, and seine nets, as appropriate. These included 18 historically present species and five whose distribution ranges fall into the study area and could be expected to occur there. In terms of counts, the most abundant species collected were *Oreochromis mossambicus* (W.K.H. Peters, 1852) (*n* = 322), *Poecilia reticulata* (W. Peters, 1859) (*n* = 244), *Enteromius trimaculatus* (W.K.H. Peters, 1852) (*n* = 172), and *Labeobarbus natalensis* (Castelnau, 1861) (*n* = 137), which accounted for 70% of all individuals captured. However, *P. reticulata* was only captured in the Mandini Stream, accounting for 68% of the relative abundance caught in that stream (Table 3). Cyprinidae was the most species-rich family in the lower uThukela River, with six indigenous species (*Enteromius paludinosus* (W.K.H. Peters, 1852), *Enteromius viviparus* (M.C.W. Weber, 1897), *E. trimaculatus*, *Labeo molybdinus* (Du Plessis, 1963), *Labeo rubromaculatus* (Gilchrist & W. W. Thompson, 1913), and *L. natalensis*) and one non-native species (*Cyprinus carpio*) (Linnaeus, 1758).

**Table 3:** A list of all fish species (excluding mullet fry and *Enteromius* spp. unidentified to species level) expected in the freshwater reaches of the study area, their historical occurrence, and presence/relative abundance in the lower uThukela River, KwaZulu-Natal, South Africa

Species	Species abbreviations	Falls in the distribution range	Historical presence	Presence (n) in the present study
*Awaous aeneofuscus*	*Aaen*	X	X	7
*Acanthopagrus berda*	*Aber*	X	X	2
*Ambassis dussumieri*	*Adus*	X		3
*Ambassis natalensis*	-	X		
*Amphilius natalensis*	-	X	X	
*Amphilius uranoscopus*	-	X	X	
*Anguilla bengalensis*	-	X	X	
*Anguilla bicolor bicolor*	-	X		
*Anguilla marmorata*	*Amar*	X	X	5
*Anguilla mossambica*	*Amos*	X	X	1
*Cyprinus carpio	*Ccar*	X	X	12
*Clarias gariepinus*	*Cgar*	X	X	48
*Coptodon rendalli*	*Cren*	X	X	
*Eleotris fusca*	*Efus*	X	X	10
*Eleotris melanosoma*	*-*	X		
*Enteromius gurneyi*	*-*	X	X	
*Enteromius paludinosus*	*Epau*	X	X	40
*Enteromius trimaculatus*	*Etri*	X	X	172
*Enteromius viviparus*	*Eviv*	X	X	58
*Enteromius *spp*. (Fry)*	*ESpp*	-	-	38
*Gilchristella aestuaria*	*Gaes*	X	X	
*Glossogobius callidus*	*Gcal*	X	X	7
*Glossogobius giuris*	*Ggiu*	X	X	1
*Hypseleotris cyprinoides*	*-*	X		
*Kuhlia rupestris*	*Krup*	X		1
*Labeo molybdinus*	*Lmol*	X	X	66
*Labeo rubromaculatus*	*Lrub*	X	X	18
*Labeobarbus natalensis*	*Lnat*	X	X	137
*Microphis brachyurus*	*Mbra*	X		2
*Microphis fluviatilis*	*Mflu*	X		1
*Micropterus salmoides	*Msal*	X		1
*Monodactylus argentus*	*-*	X		
*Monodactylus falciformis*	*-*	X		
*Mullet *spp.* (Fry)*	*Mfry*	-	-	1
*Oreochromis mossambicus*	*Omos*	X	X	322
*Pseudocrenilabrus philander*	*-*	X	X	
*Pseudomyxus capensis*	*Mcap*	X	X	54
*Poecilia reticulata	*Pret*	X	X	244
*Tilapia sparmanii*	*Tspa*	X	X	

* Non-native/invasive species

**Table 4:** Collected abundances of fish species at sample sites across the study area in the lower uThukela River, KwaZulu-Natal, South Africa

	uThukela River sites							Mandini Stream sites	
Species	Mdlebeni	EWR16	US	DS	EWR17	EWR18	EWR19	Mandini US	Mandini DS
*Aaen*		3		1	1		2		
*Aber*					1		1		
*Adus*					3				
*Amar*				3	2				
*Amos*									1
*Ccar*			2	8	1				1
*Cgar*	3		2	20	5	4	2	3	9
*Efus*				4	5				1
*Epau*	13			11	2		14		
*ESpp*		38							
*Etri*	111	18	25	7	1		6		4
*Eviv*	32	6		20					
*Gcal*		2		2	1		1		1
*Ggiu*							1		
*Krup*									1
*Lmol*		1	1	44	4				16
*Lnat*	12	9	1	33	7	42	1		32
*Lrub*		2	1	2	3		1		9
*Mbra*					2				
*Mflu*					1				
*MFry*				1					
*Msal*				1					
*Omos*		3		62	140	3	76	1	37
*Pcap*					7	13	33		1
*Pret*								180	64

Sites are listed left to right from upstream to downstream (see Fig. 1), and the shaded area is the main component of the study area around the fishway. Fish abbreviations are as per Table 3. *US* upstream, *DS* downstream, and *ESpp* unknown *Enteromius* spp., classification could not be determined because of the small size (< 10 mm TL); *MFry* mullet fry too small to identify to the species level

The proximity of the study area to the uThukela Estuary enabled the detection of euryhaline species, with *A. aeneofuscus* and *Glossogobius callidus* (J.L.B. Smith, 1937) being recorded as high up the system as site EWR16. Other euryhaline species, such as *Acanthopagrus berda* (Fabricius, 1775), *Ambassis dussumieri* (Cuvier, 1828), *E. fusca*, *Glossogobius giuris* (F. Hamilton, 1822), *K. rupestris*, *Microphis brachyurus* (Valenciennes, 1842), *Microphis fluviatilis* (Peters, 1852), and *Pseudomyxus* (formerly *Myxus*) *capensis* (Valenciennes, 1836), were only found at sites downstream of the LTBWSS weir. Four species (*Clarias gariepinus *(Burchell, 1822), *E. trimaculatus*, *L. natalensis*, and *O. mossambicus*) were widespread across the study area, except for one or two sites (Table 4). *Clarias gariepinus* was only absent at site EWR16, whereas *E. trimaculatus* could not be found at sites EWR18 and the upstream Mandini site. The only species present at all sites on the uThukela River was *L. natalensis*, except for the upstream site of the Mandini Stream, where it was absent. *Oreochromis mossambicus*, one of the study’s most abundant species (*n* = 322), was not found at two of the three upstream sites of the LTBWSS weir, Mdlebeni and UPSTR. The study sites showed varying degrees of species diversity with no clear pattern and had the following species counts on the uThukela; Mdlebeni (*n* = 5), EWR16 (*n* = 9), UPSTR (*n* = 6), DWNSTR (*n* = 15), EWR17 (*n* = 17), EWR18 (*n* = 4), EWR19 (*n* = 11). There was a significant difference in species diversity between the two sites of the Mandini Stream, with the upstream site (*n* = 3) having fewer than a quarter of the species present at the downstream site (*n* = 13). The non-native *P. reticulata* was only found in the Mandini Stream, where it was abundant at both sites, but more so at the upstream site, comprising 98% of the collected abundance (Table 4).

The preferred habitats, flow types, and migratory behaviours of the fish present and expected to be found in the study area are listed in Supplementary information Table S1. It is essential to acknowledge that the migratory classification of some potamodromous and diadromous species may not be entirely accurate, but is based on the best available literature from the South African Migratory Biota Index (Bok et al. 2007), supplemented by recent literature (O’Brien et al. 2019).

### Fish responses to environmental variables

The ‘mvabund’ package enabled us to fit multivariate fish community data to various environmental variables, including mean water velocity, ecohydraulic flow classes, dominant substrate, dominant cover features, and water quality variables, to identify which factors were driving fish communities in the study. The initial environmental variables included pH and electrical conductivity; however, a collinearity check on the model revealed an unacceptable correlation, exceeding 60%, between mean depth and velocity, as well as between electrical conductivity and total dissolved solids, electrical conductivity and pH, water temperature and pH, and total dissolved solids and pH. Therefore, electrical conductivity and pH were omitted from the model to ensure that the correlation of variables did not decrease its accuracy, while keeping depth and velocity, as both can be important drivers of local fish community structures (Kleynhans 2007), especially where flows have been altered. The full model was tested, which included eight environmental variables: ecohydraulic flow class, dominant substrate, dominant cover, mean velocity, mean depth, total dissolved solids, temperature, and salinity. Along with the main environmental variables, the levels within the environmental variables ecohydraulic flow classes (fast-deep, fast-intermediate, fast-shallow, slow-deep, and slow-shallow), dominant substrate (boulder, cobble, gravel, sand, mud, silt), and dominant cover (depth, marginal vegetation, overhanging vegetation, ripple surface, substrate, and woody debris), were also tested.

According to the results, the main environmental factors affecting the composition of the fish communities in the lower uThukela River were dominant substrate (*P* = 0.008), dominant cover (*P* = 0.028), average depth (*P* = 0.044), and water temperature (*P* = 0.008). In addition, the levels of the environmental variables showed that: for ecohydraulic flow classes, the fast-deep (*P* = 0.003) and slow-shallow (*P* = 0.006) were significant; the dominant substrate showed significance for boulders (*P* = 0.008), cobble (*P* = 0.002), gravel (*P* = 0.001), sand (*P* = 0.001), and silt (*P* = 0.015); and the dominant cover showed significance for depth (*P* = 0.001), marginal vegetation (*P* = 0.003), overhanging vegetation (*P* = 0.001), and substrate (*P* = 0.011).

**Fig. 2:**
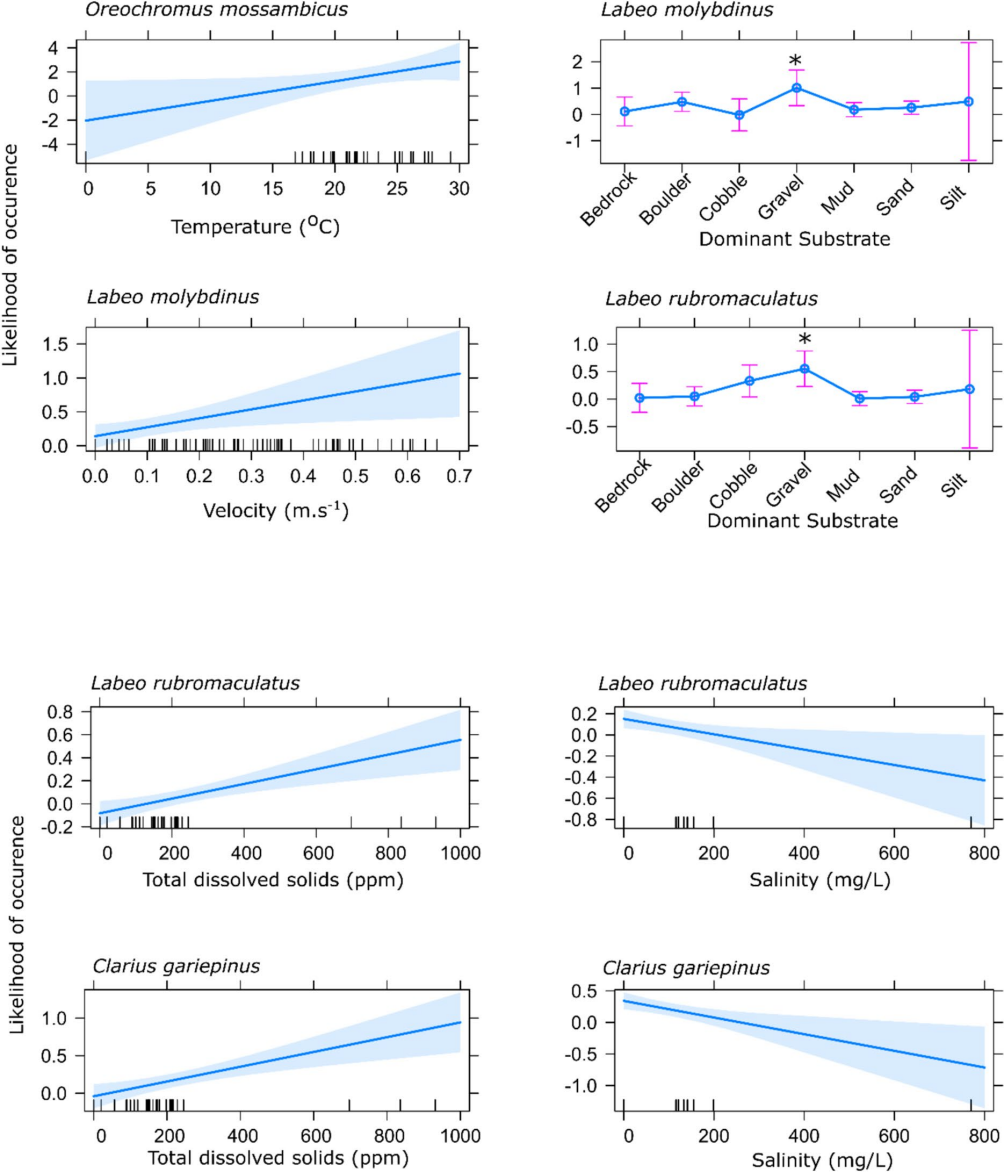
Generalised linear models showing the predictive relationship where a significant relationship exists between the likelihood of occurrence (95% confidence intervals are shaded) of ecological indicator fish species when linked to environmental variables in the lower uThukela River, KwaZulu-Natal, South Africa. (Note: Dash on the x-axis are the measured data points, and * is the significant relationship)

Five indigenous species collected in the study (*O. mossambicus*, *E. trimaculatus*, *L. natalensis*, *L. molybdinus*, *L. rubromaculatus*) were selected because of their relatively high abundances in the study and because they are known ecological indicators in the region. Here, a Generalised Linear Model (GLM) for each species against the environmental variables (ecohydraulic, dominant substrate, dominant cover, mean depth, mean velocity, temperature, total dissolved solids, and salinity), and the levels, as mentioned above, were run. Only four of these species (*O. mossambicus*, *L. molybdinus*, *L. rubromaculatus*, and *C. gariepinus*) showed significant relationships with some of the variables and are illustrated in Fig. 2. The likelihood of occurrence of *O. mossambicus* (GLM, *P* = 0.0298) was positively correlated with water temperature. The two mudfish species, *L. molybdinus* (GLM, *P* = 0.0408) and *L. rubromaculatus* (GLM, *P* = 0.0126), are substrate specialists that respond to substrate conditions, with gravel as a dominant substrate positively influencing their occurrence. In addition, for *L. molybdinus* (GLM, *P* = 0.0335), higher velocity increased the likelihood of its presence. Increases in total dissolved solids were shown to be a significant driver for both *L. rubromaculatus* (GLM, *P* = 0.0004) and *C. gariepinus* (GLM, *P* = 0.0004), with the likelihood of occurrence for both species positively correlated. The likelihood of occurrence decreased for both species, *L. rubromaculatus* (GLM, *P* = 0.0193) and *C. gariepinus* (GLM, *P* = 0.0058), when the salinity levels increased.

Less abundant species, although still important to the study, were also analysed using the same Generalised Linear Model. These were *E. viviparus, E. palludinosus, G. callidus, A. aeneofuscus*, *E. fusca*, *A. marmorata*, and *A. mossambica*. Species that showed significance to tested environmental variables were *E. fusca, G. callidus*, and *A. marmorata*. *Eleotris fusca* showed a significant relationship when environmental conditions for the ecohydraulic flow class fast-intermediate (GLM, *P* = 0.0005) and fast-shallow (GLM, *P* = 0.013) were present, as well as for the boulder substrate (GLM, *P* = 0.027) and the rippled surface as a cover feature (GLM, *P* = 0.0163). Similarly, *G. callidus* showed significant relationships with the fast-intermediate flow class (GLM, *P* = 0.0002) and silt substrate (GLM, *P* = 0.0003). The most abundant anguillid eel in the study, *A. marmorata*, showed a significant relationship to the boulder substrate (GLM, *P* = 0.0437).

## Discussion

The composition of fish communities within the lotic environment is complex, with multiple environmental variables influencing them (Carvalho and Tejerina-Garro 2015). Different fish species react differently to factors associated with habitat conditions, such as substrate and cover, as well as hydraulic parameters, including depth and velocity (Kleynhans 2007; James and King 2010). In addition, they respond to conditions associated with physical, chemical, and biological stressors, including water temperature, instream barriers, pollutants, and invasive species. According to statistical modelling, the fish communities in the present study were impacted by multiple environmental variables, with the main drivers being dominant cover, dominant substrate, depth, and temperature. Furthermore, individual species showed varying relationships to the levels of variables within these environmental drivers. The study was limited to fish species caught during the study period and focused specifically on fish community structure rather than individual fish movement. The paucity of knowledge about the timing and cues of known migratory fish species may reduce detection of these species at key upstream sites and may not align with sampling dates. In addition, erratic flows and sampling limitations during flooding conditions may reduce the detection of potentially mobile fish. The study design, at best, tried to mitigate these factors.

In other studies, geomorphic conditions, such as the dominant substrate and cover features, have been shown to significantly shape fish populations (Cheek and Taylor 2016; Desai et al. 2021). Regarding the geomorphic conditions in this study, the uThukela River and Mandini Stream differed greatly. The sites along the uThukela River were dominated by sand substrate, with a few sites featuring other substrates; the Mandini Stream had a good combination of larger substrate types (boulders and bedrock) and small-grain sediment (mud and sand). Cover features were also different, with all sites on the uThukela dominated by water-column depth, while various other cover features were present. The Mandini site was characterised by overhanging vegetation and boulders. The influence of cover and substrate was observed in the present study, with different species responding differently to the available habitat. Species with a demersal lifestyle have significant relationships to substrate conditions (Allen 1991; Skelton 2025). The two mudfish species, *L. molybdinus* and *L. rubromaculatus*, exhibit a high likelihood of occurrence in gravel substrates, among other variables, and are linked to their benthic feeding habits (Skelton 2025). Similarly, silt was a crucial substrate condition for the presence of *G. callidus* in the study, as it is suitable for organisms that live and feed on benthic organisms (Allen 1991; Skelton 2025). *Anguilla marmorata*, a species known for inhabiting rock crevices (Skelton 2025), had boulders as the primary driver of its presence in the study. In this study, *Eleotris fusca* exhibited a notable difference in its environmental drivers compared with the available literature. According to Skelton (2025), *E. fusca* is relatively inactive and found in muddy substrate conditions with logs, stones, and root wads as cover to hide under, yet in this study, they showed a preference for boulder substrate with fast intermediate and fast, shallow flow classes, an environment that creates a ripple surface cover feature. The majority of *E. fusca* individuals captured in the study were in their juvenile size class, likely in the young-of-the-year amphidromous upstream migration phase (Whitfield 2019). The detection of juvenile *E. fusca* is likely the reason our findings differ from the literature, as the species is cryptic, especially regarding our understanding of its life history. Such findings may prove valuable to the further understanding of this species.

Parameters linked to hydraulic conditions, such as depth and velocity, are also influential drivers of fish communities in lotic systems (Bice et al. 2014; Chea et al. 2020; Magoulick et al. 2021). In addition, the relationship between depth and velocity can create important ecohydraulic flow classes for fish (Kleynhans 2007; James and King 2010), as observed with juvenile *E. fusca*. Another species in the study, *G. callidus*, with a demersal lifestyle similar to *E. fusca*, also showed a preference for the fast-intermediate flow class. This preference was largely because of the detection of juvenile size classes of *G. callidus* individuals in the present study. The cyprinid, *L. molybdinus*, exhibited a positive relationship with velocity in this study, a characteristic known to be present in this rheophilic species (Desai et al. 2021; Skelton 2025). Although dominant substrate and temperature were the most significant drivers of fish communities in this study, the depth of the uThukela River partly accounts for its species diversity. Depth in a system supports diverse microhabitats for fish, as observed in other studies (Carvalho and Tejerina‐Garro 2015; Desai et al. 2021). This is likely why it is a driver of fish communities in the present study, but it did not show significance for any individual species.

Physical water conditions, such as temperature, dissolved solids, and salinity, were identified as additional drivers of fish presence in the present study. Two species, *L. rubromaculatus* and *C. gariepinus*, exhibited similar preferences for total dissolved solids and salinity, with both showing positive relationships with total dissolved solids and negative relationships with salinity, as expected for freshwater species (Skelton 2025). *Oreochromis mossambicus* showed a positive relationship with water temperature in the study area.

Biological stressors, such as non-native fish, are known to have detrimental ecological impacts on indigenous fauna in South African freshwater ecosystems (Ellender and Weyl 2014). Fortunately, in the present study and other recent studies conducted in the same area (Jacobs 2017; Evans et al. 2022), the impacts of non-native fish are low in the lower uThukela River. Non-native species, such as *C. carpio,* which are invasive species known to destroy nests of indigenous fish and out-compete them for resources (Stuart and Jones 2006), were caught in relatively low abundances in the present study. Furthermore, *M. salmoides*, an aggressive predatory fish that is dominant in the upper parts of the catchment (Evans et al. 2022; Burnett et al. 2023), where the water is less turbid, was captured in this study as a single individual. However, *P. reticulata*, a small-growing fish (< 10 cm) that is highly invasive in small rivers (Page and Burr 2011), was one of the most abundant species in this study, but was limited to the Mandini Stream. *Poecilia reticulata* is a small fish and is often preyed on by large fish when present. The uThukela River still harboured large piscivorous fish in the present study that feed on *P. reticulata*. In addition, a physical barrier is created by the weir above a natural waterfall, located 200 m upstream of the Mandini’s confluence with the uThukela, and a potential chemical barrier is created by the polluted Mandini Stream (Wade et al. 2021). The impact of the weir and poor upstream water quality on the Mandini Stream is clearly evident in the species diversity between the upstream and downstream sites (Table 4). The upstream site only had *O. mossambicus*, *C. gariepinus*, and *P. reticulata* present, of which the latter was the most abundant, compared with the downstream site, which had an additional ten species to these when it was back flooded by the high flows in the uThukela River in 2022 (Table 4; pers. obs., B. van Zyl). The presence of these three species in the Mandini is likely because of its historical function as an ephemeral system, which has recently been augmented into a perennial stream through return flows from industrial effluent and a local wastewater treatment works, abstracted from the uThukela River (Wade et al. 2021). As such, the Mandini fish assemblage comprises species with high tolerances to intermittent water supply (in its historical state) and those that can tolerate pollution. Downstream, the ten additional species observed in 2022 exhibit varying sensitivities to water pollution and migrate in and out of the uThukela River.

The impact of the LTBWSS weir on the fish community composition can be observed by comparing euryhaline species captured at different sites upstream and downstream of it, as well as the general species richness on either side. The three upstream sites (Mdlebeni, EWR16, UPSTR) were dominated by purely freshwater fish, with only two species of euryhaline fish (*A. aeneofuscus* and *G. callidus*) found at site EWR16, which also had the highest upstream diversity of nine species (Table 4). This is compared with the downstream sites, which had a combined total of 12 euryhaline species, including five at the immediate downstream site (DWNSTR) and nine at site EWR17, which was also the most diverse site in the study, with 17 species (Table 4). The diversity at this site is likely because of its location directly below the Paper Mill abstraction point, which serves as a partial barrier formed by a natural dyke across the river. This dyke was excavated to enhance water abstraction by the Paper Mill, creating a partial barrier, where migrating species may congregate. The difference in euryhaline species between these two sites is likely because of a partial barrier created by the Paper Mill extraction point located between them (Jacobs 2017; Wade et al. 2021). Furthermore, a direct comparison between the sites immediately upstream (UPSTR) and downstream (DWNSTR) showed the weir’s impacts on their fish communities.

The UPSTR site is a deep impoundment with depth primarily serving as cover for fish and was dominated by a mud substrate, with low species richness (*n* = 6). Unlike DWNSTR, which was dominated by boulders and a sand substrate, it had various depth profiles and boulders as cover, and demonstrated much higher species richness (*n* = 15). Additionally, the long-term temporal effects that the LTBWSS may have had on downstream fish communities were compared with those reported by Jacobs (2017). Their study was conducted on a 4 km stretch downstream from the weir location before it was constructed and included a fish collection component using sampling methods similar to those employed in the present study (seine netting, electrofishing, and cast netting). Notably, their study collected all four expected cichlid species (*Coptodon rendalli*, *O. mossambicus*, *Pseudocrenilabrus philander*, *T. sparrmanii*) at relatively high abundances, with *T. sparrmanii* having the most individuals collected (*n* = 550) (Jacobs 2017). Subsequently, their study found that *T. sparrmanii* showed a strong correlation with silt substrate, particularly among juveniles, and that their community structures changed significantly when substrate conditions changed (Jacobs 2017). In contrast, our study detected only *O. mossambicus*, despite sampling the same sites. Such a significant shift in cichlid populations is concerning, especially considering that they are a crucial food source for many local subsistence fisheries (Coetzee et al. 2015). Cichlids require sandy substrates for creating breeding beds. It is possible that the LTBWSS weir acts as a sediment trap, reducing the downstream delivery of these important sediments (Casserly et al. 2021). In addition, reduced water flow from abstraction compounds the effects of downstream pollution from both the Mandini Stream and the Paper Mill (Wade et al. 2021). Site EWR18 had the lowest species richness in the uThukela River (*n* = 4), as shown, resulting from flow alterations and sedimentation behind the LTBWSS weir. Poor water quality could also explain why *T. sparmanii* and *C. rendalii* would be absent (Desai et al. 2021; Evans et al. 2022).

## Conclusions and recommendations

Our findings provide a baseline ecological understanding of the fish community in the lower uThukela catchment post-development of the LTBWSS Weir, and we identified significant environmental determinants of the fish community composition in the region. The inclusion of ad hoc sites in the study helped provide a broader perspective on fish community structures and the drivers of the entire lower uThukela system. Different environmental variables influence the presence of various fish species in the region, thereby affecting the overall fish community composition across sites. This indicates that the LTBWSS presently reduces the occurrence of euryhaline species upstream.

Our study demonstrated that the LTBWSS weir affects fish communities upstream and downstream of it. Upstream communities were predominantly freshwater species, with only *G. callidus* considered euryhaline. The upstream impact appears to be minimal; however, *P. capensis* and *K. rupestris*, which are known to move upstream into freshwater systems, were not found upstream of the LTBWSS weir. The study did not detect *G. aestuaria*, which may be confined to the estuary, although it has been recorded historically. Therefore, downstream communities are expected to be more species diverse. The LTBWSS weir and modifications to enhance the Paper Mill abstraction point (Wade et al. 2021) may occur at critical points between the estuary and the lower uThukela, potentially limiting the upstream movement of key diadromous species beyond site EWR16. The lack of freshwater species, such as cichlids, is concerning. It is likely that downstream of the LTBWSS weir does not receive transportation of the required fine sediments, which were crucial to the cichlid species *T. sparrmanii,* which was found in the region in very high abundances prior to the construction of the weir, but were not recorded during this study, despite extensive sampling. Additionally, the increased abstraction of water by the LTBWSS infrastructure reduces downstream flows into the estuary, compounding the impacts of pollution sources from the Mandini Stream and the Paper Mill effluent on site EWR18.

The scarcity of data regarding when and where euryhaline species migrate between freshwater and estuarine habitats is a concern. These data are hard to collect; future research should specifically focus on species with relatively unknown life histories by collecting fish movement data, which will help better understand their habitat preferences, migratory behaviours, and life cycles. These are important considerations for monitoring their well-being in a system. The cichlid not detected in this study needs consideration in how the LTBWSS is managed, with improved resource management and conservation practices, as this could allow these species to regenerate. The newly constructed LTBWSS has a measurable impact on the downstream fish communities, impacting people’s livelihoods and ecological well-being. The management of the LTBWSS needs to consider these impacts and mitigate against them to improve the ecological conditions of the lower uThukela River. Lastly, the efficacy of the fish ladder installed in the LTBWSS Weir in maintaining the movement of migratory fish requires further investigation.

## Supplementary information

Below is the link to the electronic supplementary material.

ESM 1 (DOCX 33.3 KB)

## Data Availability

The data belong to the University of KwaZulu-Natal and are available from the authors upon reasonable request.
