# Supplementary material for: A fragmented fish community: evaluation of the present distribution and drivers of fish communities in the lower uThukela River, KwaZulu-Natal, South Africa
Source: Environ Biol Fishes. 2026 Mar 4;109(1):54. doi: 10.1007/s10641-026-01822-y (PMC12960327; doi:10.1007/s10641-026-01822-y)
Supplement: Supplementary file 1 — (DOCX 33.3 KB) [file 10641_2026_1822_MOESM1_ESM.docx]

**Supplementary information**

**Supplementary information Table S1**: A list showing the preferred habitats and flow types, migratory behaviour, and IUCN status of native and non-native fish species found in the uThukela River catchment, KwaZulu-Natal Province, South Africa (sourced from the South African Migratory Biota Index (Bok et al. 2007), and supplemented by recent literature (e.g., O’Brien et al. 2019)).

|  | **Species** | **Abbr.** | **Preferred habitat and flow types** | **Migratory behaviour** | **IUCN Status** |
| --- | --- | --- | --- | --- | --- |
| 1 | *Awaous aeneofuscus*  **Freshwater Goby**  (Peters, 1852) | AAEN | Benthopelagic, Rivers and estuaries. Quiet swift water, also lagoons. (Skelton 2001) | Anadromous (Bok et al. 2007), possibly Amphidromous (Whitfield 2019) | LC |
| 2 | *Acanthopagrus berda*  **River bream**  (Forskall, 1775) | ABER | Demersal. Marine, Freshwater, Brackish. Spawn in estuaries (Garratt 1993). | Oceonodromous (Riede 2004), Amphidromous (Bok et al. 2007) | LC |
| 3 | *Anguilla bengalensis labiata*  **African mottled eel**  (Peters. 1852) | ABEN | Benthopelagic, rock pools. Streams, pools and reservoirs (Skelton 2001). | Catadromous. | NT |
| 4 | *Anguilla bicolor bicolor*  **Shortfin eel**  (McClelland, 1844) | ABIC | Demersal. Freshwater as an adult, estuaries and seas as young. Rocky bottoms and deep pools, prefers marshes. Predominantly lowland rivers. Freshwater streams and pools. (Skelton 2001) | Catadromous | NT |
| 5 | *Anguilla marmorata*  **Giant mottled eel**  (Quoy & Gaimard, 1824) | AMAR | Demersal, freshwater as adults, estuaries and seas as young, rocky bottoms and deep pools, throughout the river into upland tributaries. Freshwater streams and pools. (Skelton 2001) | Catadromous | LC |
| 6 | *Anguilla mossambica*  **African longfin eel**  (Peters, 1852) | AMOS | Demersal, freshwater as adults, estuaries and seas as young, rocky bottoms and deep pools, throughout the river into upland tributaries. Streams and pools. (Skelton 2001). | Catadromous | NT |
| 7 | *Amphilius natalensis*  **Natal mountain catfish**  (Boulenger, 1917) | ANAT | Demersal, freshwater. Cobbles and rocks of swiftly flowing streams. Common in upland streams. (Skelton 2001) | Potamodromous | LC |
| 8 | *Amphilius uranoscopus*  **Stargazer mountain catfish**  (Pfeffer, 1889) | AURA | Demersal, freshwater. Rocky habitats. Clear, flowing water. (Skelton 2001) | Potamodromous | LC |
| 9 | *Ambassis dussumieri*  **Bald glassy**  (Cuvier, 1828) |  | Demersal, marine to freshwater. Tolerant of freshwater within a narrow temperature range (23 – 26 C) | Unknown, possibly diadromous of sorts | LC |
| 10 | *Ambassis natalensis*  **Slender glassy**  (Gilchrist & Thompson, 1908) |  | Demersal, marine to freshwater. Tolerates freshwater within a temperature range of 19 – 27 C. | Unknown, possibly diadromous of sorts | LC |
| 11 | **Cyprinus carpio*  **Common carp**  (Linnaeus, 1758) | CCAR | Benthopelagic, freshwater, brackish. Well-vegetated lakes. Soft bottom sediments. Warm, deep, slow-flowing waters.  Vulnerable in its native range, considered an invasive elsewhere. (Kottelat and Freyhof 2007) | Potamodromous. Considerable spawning migrations to backwaters and flooded meadows. (Kottelat and Freyhof 2007) | VU |
| 12 | *Clarias gariepinus*  **Sharptooth catfish**  (Burchell, 1822) | CGAR | Benthopelagic, freshwater. Prefer shallow and swampy areas with a soft muddy substrate. May also occur in fast-flowing rivers and rapids. Quiet waters, lakes and pools. (Teugels 1986; Skelton 2001) | Potamodromous. Migrate to rivers and temporary streams to spawn. | LC |
| 13 | *Coptodon rendalli*  **Redbreast tilapia**  (Boulenger, 1897) | CREN | Benthopelagic, freshwater, brackish. Prefers quiet, well-vegetated water along river banks. Backwaters, floodplains and swamps. (Skelton 2001) | Potamodromous | LC |
| 14 | *Eleotris fusca*  **Dusky sleeper**  (Forster, 1801) | EFUS | Demersal, marine, freshwater, brackish. Lagoons, estuaries and lower reaches of freshwater streams and prefer muddy bottoms (Skelton 2001). Juveniles are mainly found in more saline environments of lagoons and estuaries, with adults also found in freshwater (Pethiyagoda 1991). | Amphidromous | LC |
| 15 | *Eleotris melanosoma*  **Broadhead sleeper**  (Bleeker, 1852) | EMEL | Demersal, marine to freshwater. Muddy reaches of estuaries and mangrove swamps, sometimes entering freshwater in medium to large rivers. Also found amongst bank vegetation of freshwater streams. (Skelton 2001) | Amphidromous | LC |
| 16 | *Enteromius anoplus*  **Chubbyhead barb**  (Weber, 1897) | EANO | Benthopelagic. Freshwater, brackish. Favours cooler waters. Various habitats, from small streams to large rivers and lakes. Associated with fallen logs, brushwood, or marginal vegetation habitats. (Skelton 2001) | Potamodromous. Lateral migrations to flooded banks after rains for breeding purposes. (Skelton 2001) | LC |
| 17 | *Enteromius gurneyi*  **Redtail barb**  (Gunther, 1868) | EGUR | Benthopelagic. Freshwater. Small streams in the sandstone belt. Favours pools, where it is frequently the only other fish species besides freshwater eels. (Skelton 2001) | Potamodromous | VU |
| 18 | *Enteromius paludinosus*  **Straightfin barb**  (Peters, 1852) | EPAU | Benthopelagic. Freshwater. Prefers quiet, well-vegetated waters in lakes, swamps, and marshes or marginal areas of large rivers or slow-flowing streams. (Skelton 2001) | Potamodromous. Moving into flooded vegetation and influent rivers during the rainy season for spawning. (Skelton 2001) | LC |
| 19 | *Enteromius trimaculatus*  **Threespot barb**  (Peters, 1852) | ETRI | Benthopelagic. Freshwater. Found in shallow waters near river outlets, also close to swampy areas. Various habitats, especially where there is vegetation (Skelton 2001). Thrives in sand and rock-bottomed streams and can tolerate degraded habitats and streams. | Potamodromous. Upstream movements in flooded rivers after rains for breeding (Skelton 2001). | LC |
| 20 | *Enteromius viviparus*  **Bowstripe barb**  (Weber, 1897) | EVIV | Benthopelagic. Freshwater. Vegetated pools of streams and rivers and lake margins, usually in the Lowveld, coastal plains (Skelton 2001). Relies on submerged roots and vegetation to lay eggs on. | Potamodromous | LC |
| 21 | *Gilchristella aestuaria*  **Gilchrist’s round herring**  (Gilchrist, 1913) | GAES | Pelagic-neritic. Marine, freshwater, brackish. Mostly found in estuaries, and also rivers and lakes (Whitfield 1998). | Unknown | LC |
| 22 | **Gambusia affinis*  **Mosquitofish**  (Baird & Girard, 1853) | GAFF | Benthopelagic. Freshwater, brackish. Most abundant in lower reaches of streams. Adults inhabit standing to slow-flowing water, most common in vegetated lakes, backwaters, and quiet pools of streams (Page and Burr 2011). | Potamodromous | LC |
| 23 | *Glossogobius callidus*  **River goby**  (Smith, 1937) | GCAL | Benthopelagic. Freshwater, brackish. Inhabits rivers and the upper reaches of estuaries. Lives in pools, on the bottom amongst cobbles or vegetation (Whitfield 1998). Although found in estuaries, larvae were most abundant in mesohaline regions (Strydom and Neira 2006). | Unknown, although literature may suggest amphidromous with larvae found in estuaries (Strydom and Neira 2006), most likely from larval drift by river currents similar to *G. giuris* | LC |
| 24 | *Glossogobius giuris*  **Tank goby**  (Hamilton, 1822) | GGIU | Benthopelagic. Marine, freshwater, brackish. Mainly freshwater and estuaries. Found in clear to turbid streams, usually with rock, gravel, or sand bottoms (Allen 1991). | Amphidromous. Spawning in freshwater, relies on larval drift by river currents to wash eggs and larvae into the sea (Allen 1991). | LC |
| 25 | *Hypseleotris cyprinoides*  **Golden sleeper**  (Valenciennes. 1837) | HCYP | Demersal, marine to freshwater. Favours shallow vegetated margins in freshwater streams that enter estuaries. Threatened by coastal development and habitat destruction (Skelton 2001). | Amphidromous | DD |
| 26 | **Lepomis macrochirus*  **Bluegill**  (Rafinesque, 1819) | LMAC | Benthopelagic. Freshwater. Frequently found in lakes, ponds, reservoirs and sluggish streams. Prefers deep weed beds. (Page and Burr, 2011) | Unknown. | LC |
| 27 | *Labeo molybdinus*  **Leaden labeo**  (du Plessis, 1963) | LMOL | Benthopelagic. Freshwater. Prefers rapids but is absent from the coldest streams. Often in large permanent pools of large rivers and will enter rapids. Rocky habitats of the main river channel (Skelton 2001). | Potamodromous. Upstream migrations for breeding in swollen rivers after rains (Skelton 2001). | LC |
| 28 | *Labeobarbus natalensis*  **KwaZulu-Natal yellowfish**  (Castelnau, 1861) | LNAT | Benthopelagic. Freshwater. Wide variety of habitats, from pools and rapids of clear streams to deep turbid waters of rivers and impoundments (Burnett et al. 2021b). Prefers warmer areas of rivers, often congregating at inlets of small tributaries. Spawns in fast-flowing stretches of river, over algae-free gravel beds (Skelton 2001). | Potamodromous. Upstream migrations in spring and summer for feeding and breeding purposes (Skelton 2001). Downstream migration to deep pools in winter (Burnett et al., 2021b). | LC |
| 29 | *Labeo rubromaculatus*  **Tugela labeo**  (Gilchrist & Thompson, 1913) | LRUB | Benthopelagic. Freshwater. Endemic to the uThukela catchment. Occurs from sea level up to an elevation of 1520m. Prefers deep pools and slow-flowing rivers and also occurs in rocky rapids (Skelton 2001). | Potamodromous. Upstream spring and summer migration for breeding (Skelton 2001). | VU |
| 30 | *Monodactylus argenteus*  **Round moony**  (Linnaeus, 1758) | MARG | Pelagic-neritic. Marine, freshwater, brackish. Found in bays, tidal creeks, mangrove estuaries, and lower freshwater reaches of rivers (Allen 1991). Often frequenting vegetation with juveniles entering freshwater (Skelton 2001). | Unknown. | LC |
| 31 | *Monodactylus falciformis*  **Oval moony**  (Lacepede, 1801) | MFAL | Marine, freshwater, brackish. Coastal waters, as well as estuaries and lagoons for juveniles. Associated with reefs. Often frequenting vegetation (Skelton 2001). | Catadromous (Bok et al. 2007) | LC |
| 32 | *Microphis brachyurus*  **Short-tailed pipefish**  (Bleeker, 1854) | MBRA | Demersal. Marine, freshwater, brackish. Relatively shallow, still to slow-flowing water (Pethiyagoda, 1991). Juveniles and subadults are usually found in estuaries, while adults are found upstream in freshwater. | Anadromous. | LC |
| 33 | *Microphis fluviatilis*  **Freshwater pipefish**  (Peters, 1852) | MFLU | Demersal. Marine, freshwater, brackish. Coastal rivers and streams in quiet water amongst vegetation or logs at river edges (Okeyo 1998). | Unknown, possibly anadromous like *M. brachyurus* | DD |
| 34 | **Micropterus dolomieu*  **Smallmouth bass**  (Lacepede, 1802) | MDOL | Benthopelagic. Freshwater. Shallow rocky areas of lakes and clear, gravel-bottom runs and flowing pools of rivers. Shallow sand, gravel, or rocky bottoms for nesting (Page and Burr 2011). | Potamodromous | LC |
| 35 | **Micropterus punctulatus*  **Spotted bass**  (Rafinesque, 1819) | MPUN | Demersal. Freshwater. Clear to slightly turbid, gravel-bottomed and flowing pools. Runs and creeks and small to medium rivers. Also inhabits lakes and reservoirs (Page and Burr 2011). | Potamodromous. | LC |
| 36 | **Micropterus salmoides*  **Largemouth bass**  (Lacepede, 1802) | MSAL | Benthopelagic. Freshwater. Inhabit lakes, ponds, swamps, backwaters, pools of creeks, and small to large rivers. Prefers quiet or slow-flowing, clear water with submerged and floating vegetation and overgrown banks (Page and Burr 2011). | Potamodromous. | LC |
| 37 | *Oreochromis mossambicus*  **Mozambique tilapia**  (Peters, 1852) | OMOS | Benthopelagic. Freshwater, brackish. Thrives in standing waters. Commonly over mud bottoms, often in well-vegetated areas (Skelton 2001). Common in blind estuaries and coastal lakes but usually absent from permanently open estuaries, and fast-flowing water, as well as at high altitudes (De Moor and Bruton 1988). Can grow and reproduce in fresh and brackish water. | Amphidromous in estuarine and coastal lakes. Potamodromous in freshwater (Riede 2004; Bok et al. 2007). | VU |
| 38 | **Oncorhynchus mykiss*  **Rainbow trout**  (Walbaum, 1792) | OMYK | Benthopelagic. Freshwater only in South Africa. Inhabit clear, cold headwaters, high-altitude rivers and lakes. Stocked in lakes. Cool (<21°C), well-aerated water is necessary. Needs gravel-bottomed beds for breeding (Skelton 2001; Page and Burr 2011). | Potamodromous in South Africa. Upstream migration to clear headwaters for breeding (Skelton 2001). | NE |
| 39 | *Pseudomyxus capensis*  **Freshwater mullet**  (Valenciennes, 1836) | PCAP | Demersal. Marine, freshwater, brackish. Found as far upriver as 135km from the mouth in South Africa (Bok 1979). | Catadromous. Breeding at sea, juveniles move into estuaries and rivers for growth and maturing (Skelton 2001). | LC |
| 40 | *Pseudocrenilabrus philander*  **Southern mouthbrooder**  (Weber, 1897) | PPHI | Benthopelagic. Freshwater. From flowing waters to lakes, usually prefers vegetated zones where the current is not too strong (Skelton 2001). | Potamodromous. Upstream migrations during heavy rains. | LC |
| 41 | **Poecilia reticulata*  **Guppy**  (Peters, 1859) | PRET | Benthopelagic. Freshwater, brackish.  Inhabits warm springs, weedy ditches and canals. Various habitats from turbid water in ponds, canals, and ditches at low elevation to high altitude mountain streams. Vegetation is essential (Page and Burr 2011). | Non-migratory. | LC |
| 42 | **Salmo trutta*  **Brown trout**  (Linnaeus, 1758) | STRU | Pelagic-neritic. Freshwater only in South Africa. Inhabit clear, cold headwaters, high-altitude rivers and lakes. Stocked in lakes. Cool (<21°C), well-aerated water is necessary. Needs gravel-bottomed beds for breeding (Skelton 2001, Page and Burr 2011). | Potamodromous. Upstream migration to clear, flowing headwaters for breeding. (Skelton 2001) | LC |
| 43 | *Tilapia sparmanii*  **Banded tilapia**  (Smith, 1840) | TSPA | Benthopelagic. Freshwater. Found in various habitats, favours areas with submerged or emergent vegetation along edges of rivers, lakes, and swamps (Skelton 2001). Tends to be confined to shallow weedy areas. Spawns on substrate or branches of aquatic weeds. | Potamodromous. Seasonal upstream migrations, breeding before and during them. | LC |

*Non-native species

**Supplementary Information References**

Allen GR (1991) Field guide to the freshwater fishes of New Guinea. Christensen Research Institute, Lexington, MA.

Bok A, Kotze P, Heath R (2007) Guidelines for the planning, design and operation of fishways in South Africa, Water Research Commission, Pretoria.

Burnett MJ, O’Brien GC, Jewitt G, Downs CT (2021) Temporal and spatial ecology of an iconic *Labeobarbus* spp. in a socio-economically important river. Environ Biol Fishes 104:1103-1119.

De Moor IJ, Bruton MN (1988) Atlas of alien and translocated indigenous aquatic animals in southern Africa. National Scientific Programmes Unit, CSIR, Pretoria.

Garratt P (1993) Spawning of riverbream, *Acanthopagrus berda*, in Kosi estuary. Afr Zool 28:26-31.

Kottelat M, Freyhof J (2007) Handbook of European freshwater fishes, Publications Kottelat.

O’Brien GC, Ross M, Hanzen C, Dlamini V, Petersen R, Diedericks GJ, Burnett MJ (2019) River connectivity and fish migration considerations in the management of multiple stressors in South Africa. Marine Freshwater Res 70:1254-1264.

Okeyo DO (1998) Updating names, distribution and ecology of riverine fish of Kenya in the Athi-Galana-Sabaki River drainage system. Naga, ICLARM Quarterly, Jan-Mar, 44-53.

Page LM, Burr BM (2011) Peterson field guide to freshwater fishes of North America north of Mexico, Houghton Mifflin Harcourt.

Pethiyagoda R 1991. Freshwater fishes of Sri Lanka. Wildlife Heritage Trust of Sri Lanka.

Riede K (2004) Global register of migratory species: from global to regional scales: final report of the R&D-Projekt 808 05 081, Federal Agency for Nature Conservation.

Skelton P (2001) A Complete Guide to the Freshwater Fishes of Southern Africa. Struik Publishers, Cape Town, South Africa.

Strydom NA, Neira FJ (2006) Description and ecology of larvae of *Glossogobius callidus* and *Redigobius dewaali* (Gobiidae) from temperate South African estuaries. Afr Zool 41:240-251.

Teugels GG (1986) A systematic revision of the African species of the genus *Clarias* (Pisces; Clariidae). Annales-Musee Royal de l'Afrique Centrale. Sciences Zoologiques (Belgium).

Whitfield AK (2019) Fishes of Southern African estuaries: From species to systems, Smithiana Monograph No. 4, J.L.B. Smith Institute of Ichthyology, Grahamstown.
